# The Effect of External Apple Vinegar Application on Varicosity Symptoms, Pain, and Social Appearance Anxiety: A Randomized Controlled Trial

**DOI:** 10.1155/2016/6473678

**Published:** 2016-01-03

**Authors:** Derya Atik, Cem Atik, Celalettin Karatepe

**Affiliations:** ^1^Nursing Division, School of Health, Osmaniye Korkut Ata University, Osmaniye, Turkey; ^2^Department of Cardiovascular Surgery, Private New Life Hospital, Osmaniye, Turkey; ^3^Department of Cardiovascular Surgery, Mustafa Kemal University, Hatay, Turkey

## Abstract

*Aim*. We aimed to determine the effect of external apple vinegar application on the symptoms and social appearance anxiety of varicosity patients who were suggested conservative treatment.* Method*. The study was planned as an experimental, randomized, and controlled study. 120 patients were randomly selected and then were randomly allocated to either experimental or control group by simple blind random sampling method. In the collection of research data, a questionnaire questioning sociodemographic and clinical characteristics, the Visual Analog Scale (VAS) for pain, and the Social Appearance Anxiety Scale (SAAS) were used. The patients in the study group were suggested to apply apple vinegar to the area of the leg with varicosity alongside the treatment suggested by the doctor. The patients in the control group received no intervention during the study.* Results*. The sociodemographic and clinic characteristics of both groups were found to be similar (*p* > 0.05). The patients were evaluated with regard to cramps, pain, leg fatigue perception, edema, itching, pigmentation, and weight feelings in the leg, VAS, and SAAS averages in the second evaluation; the control group had a decrease in such symptoms (*p* > 0.05) although the decrease in the application group was higher and statistically meaningful (*p* < 0.05).* Conclusion*. We determined that the external application of apple vinegar on varicosity patients, which is a very easy application, increased the positive effects of conservative treatment.

## 1. Introduction

According to the American National Institute of Health, complementary and alternative medicine (CAM) is a wide field of health encompassing all of the health services, methods, applications, and accompanying theory and beliefs that are outside of the politically prevalent health system in a certain society or culture in any given time period [1]. Integrative medicine, which holistically evaluates CAM and evidence based medical applications, is different from CAM. First, while CAM appliers apply treatment mostly instead of scientific medicine and sometimes alongside it, integrative medicine appliers apply complementary medicine treatments in a manner compliant with scientific medicine [2]. In developed countries, 30 to 50% of the adult population prefer CAM methods to prevent or treat health problems [3]. In our country, with the recent increase in demand for CAM, regulations regarding these methods were prepared by the Ministry of Health [4]. Thus, the CAM methods that have been used for centuries and still attract public attention were aimed to be performed in hospital settings or clinics instead of unhealthy conditions.

Venous failure and induced lower extremity varicosity are an important health problem that is seen frequently in the society that decreases quality of life and can cause serious complications under certain circumstances. Lower extremity varicosity was found to be encountered in 25 to 50% of the adult population in different studies [5–7]. One of the most important complaints of varicosity patients is serious aesthetic concerns. Varicosity is a problem frequently encountered by all health personnel. The main goal in lower extremity varicosity treatment must be to remove the complaints of the patient such as pain, edema, cramps, irritability, fatigue, itching, tingling, pigmentation, burning, feeling of weight, bleeding, and ulceration (if present) by taking into account aesthetic concerns [8].

According to the National Health Interview Survey, natural products come first among the most widely used CAM methods [9]. Even though plants or plant based natural products do not remove varicosity entirely, the effects they have can reduce symptoms, reduce complication development, or reduce aesthetic concerns. When used in a correct manner alongside medical control and treatment, the foremost natural plant based products that can be used are apple vinegar, garlic,* Aloe vera*, green clay, chili peppers, chestnut tree bark, lemon oil, mustard oil, and horse chestnut seeds. Apple vinegar, which is known to have healing properties, is stressed in many websites to cause improvement in varicosity symptoms and appearance and is reported to be widely used among the public [10, 11]. Today, especially with the internet becoming more widespread, a net providing information and sales opportunities for this field has formed. However, because of the information obtained through these nets being missing and/or incorrect, individuals may face undesired consequences. For this reason, evidence from well-designed studies is necessary. When the literature was examined, no scientific studies examining the effect of apple vinegar application on varicosity were found. Nurses and other health professionals who adopt a holistic approach to patient care should have information regarding the effects, side effects, and reliability of CAM methods.

*Aim*. We aimed to determine the effect of external apple vinegar application on the symptoms and social appearance anxiety of varicosity patients who were suggested conservative treatment.

*Hypotheses*

1. Symptoms will reduce with greater effect in varicosity patients who had apple vinegar applied externally alongside conservative treatment with regard to patients who only had conservative treatment applied.
2. Social appearance anxiety will reduce with greater effect in varicosity patients who had apple vinegar applied externally alongside conservative treatment with regard to patients who only had conservative treatment applied.

## 2. Method

The study was planned as an experimental, randomized, and controlled study.

### 2.1. Sample Selection

The study was performed between August 2015 and November 2015 in the Private Yeni Hayat Hospital Cardiovascular Surgery Polyclinic. In sample selection the power analysis, which is a method ensuring the reliability, validity, and accuracy of research results, was used. The pattern of the study consisted of the apple vinegar application group and the control group which received no such intervention. Since there were no similar studies, a power analysis was performed taking the first 18 patients in both the application and control groups as a basis, and it was determined as a result that 74 patients were required to perform the study with 90% power. Since there could be sample losses during study, the study was initiated with 80 patients with 40 in each group. As a result of the power analysis performed at the end of the study, the study was determined to be performed with 100% power.

The inclusion criteria for the patients were having a CEAP classification between 1 and 3, being suggested only conservative treatment, being 18 years of age and above, having no communication problems, being of sufficient capability to answer all of the questions, agreeing to interviews, and speaking Turkish.

Randomization was performed by a different researcher blind to personal and clinical characteristics using a randomization schematic with a simple randomization method according to the registry sequence of the patients to the polyclinic.

### 2.2. Measures

In the collection of research data, a questionnaire questioning sociodemographic and clinical characteristics, the VAS, and the Social Appearance Anxiety Scale (SAAS) were used.

The Social Appearance Anxiety Scale (SAAS) is a self-report, 5-way Likert type, 16-item scale developed by Hart et al. (2008) in order to measure emotional, cognitive, and behavioral anxiety individuals experience regarding their appearance. The items were scored from (1) not appropriate at all to (5) completely appropriate. The Turkish validity and reliability of the scale were performed by Doğan (2010). A higher score in the scale is evaluated as a sign of decreasing self-respect [12, 13].

The VAS was used to measure pain intensity. In VAS, the patient places the level of pain he/she experiences between the 0 left end (no pain) and 10 right end (the most intense pain experienced) on a simple 10 cm horizontal line. The VAS can be easily used by patients and can be used over and over to monitor the response of a patient to a therapeutic intervention.

In the questionnaire for sociodemographic and clinical characteristics, the gender, age, educational status, occupation, height, weight, family history, CEAP classification, tobacco and alcohol use, HT, DM, and information on varicosity symptoms were questioned.

### 2.3. Study Procedures and Data Collection

Patients who were diagnosed with varicosity in the cardiovascular surgery polyclinic that met the criteria were informed about the study and given the questionnaire questioning sociodemographic and clinical characteristics, pain scale, and the SAAS (pretest).

The patients in the study group were suggested to apply apple vinegar to the area of the leg with varicosity alongside the treatment suggested by the doctor. The patients were taught to elevate legs by 45 degrees, moisten a cotton or rag piece with natural apple vinegar, apply this to the area with varicosity (by wrapping), and wait for 30 minutes twice a day in the morning and evening for a month. Before apple vinegar application, patients were asked to apply apple vinegar to a small part of the leg beforehand to determine the presence of allergies. In the case of dryness being present on the leg because of the application, the patients were told to apply moisturizer to the area and abandon the application in case of irritation on the skin. Allergies or irritation was not seen on any of the patients during or after the study.

The patients in the study group were called via phone each week to evaluate their application status. The patients in the control group received no intervention during the study. At the end of the one-month application process, the patients in both groups were called to the hospital for evaluation and given the questionnaire, pain scale, and SAAS again (posttest). Data was collected via face to face interviews and patient files.

### 2.4. Ethical Aspect

In the progression of the study, scientific principles as well as the ethical principles of the Helsinki Declaration were held. In this context, the principles of informed consent, autonomy, secrecy, and the protection of secrecy, fairness, and no harm were taken into consideration. Necessary written permissions from the necessary institutions were taken. In order to conduct the study, the written permission and approval of the ethics committee were received. Before the application, patients were shown the aim, plan, and benefits of the study. Informed consent was taken from the patients.

### 2.5. Analysis

Data was evaluated using the SPSS 21.0 (SPSS, Inc., Chicago, IL, USA) statistics program. Continuous variables are used mean ± standard deviation and numbers (percentages). For categorical variables between the two groups in the evaluation chi-square test, independent samples *t*-test and the paired sample *t*-test were used.

## 3. Results

The sociodemographic characteristics of the patients in the application and control groups were given in Table 1. The characteristics of both groups were found to be similar (*p* > 0.05). Most of the patients were married, housewives, or laborers. More than half of the patients had positive family histories (Table 1).

The clinical characteristics of the patients were given in Table 2. When the BMI of the patients was examined, most of the patients in both the application (67.5%) and control (67.5%) were seen to be overweight or obese. No difference was found between the groups regarding clinical characteristics (*p* > 0.05) (Table 2).

Symptoms before and after apple vinegar application were given in Table 3. Even though statistically meaningful decreases in pain and leg fatigue perceptions were seen in both groups (*p* < 0.05) the reduction in the application group was seen to be higher. When the patients were evaluated with regard to cramps, edema, itching, pigmentation, and weight feelings in the leg in the second evaluation, the control group had a decrease in such symptoms (*p* > 0.05) although the decrease in the application group was higher and statistically meaningful (*p* < 0.05) (Table 3).

The pain perception and social appearance anxiety score averages of the patients were shown in Table 4. Statistically meaningful decreases in the score averages of both groups were found (*p* < 0.05). However, the decrease in the application group was seen to be higher with a better level of statistical meaningfulness (Table 4).

## 4. Discussion

The influence of apple cider vinegar has been investigated for hundreds of years. In fact it was first used about 5000 years ago. In the year 400 B.C., Hippocrates, the father of modern medicine, prescribed the mixture of honey and apple cider vinegar for treatment of various diseases. It has been particularly used during the American Civil War for disinfecting the wounds of soldiers [14].

Vinegar is produced from fruit juices such as grape, apple, plum, coconut, and tomato, rice, and potato. It is made by crushing apples and squeezing out the liquid. Bacteria and yeast are added to the liquid to start the alcoholic fermentation process, and the sugars are turned into alcohol. In a second fermentation process, the alcohol is converted into vinegar by acetic acid-forming bacteria (*Acetobacter*) [15].

Vinegar is a plant based product that has been known and used for unknown years. Vinegar has many proven positive effects on health such as an antibacterial effect, reduction in blood pressure, an antioxidant effect, an antidiabetic effect, an antitumor effect, reduction and prevention of obesity, an antihypertensive and cholesterol decreasing effect, a healing effect on injuries, and a positive effect on brain and cognitive functions [16–25].

Apple products are widely used in the world. Nevertheless, scientific information about the biological effects of apple cider vinegar as a traditional medicine is inadequate. More valuable properties of apple cider vinegar, its ingredients, and also their therapeutic effects have been recently discovered [26–29].

Apple cider vinegar contains polyphenolic compounds that have beneficial health effects [29, 30]. It is used not only as a seasoning but also as a common a traditional medicine [20]. Varicosity has negative effects on the quality of life of patients, and it causes important workforce losses [31, 32]. The aim of conservative treatment in varicosity patients is to reduce the symptoms of the condition, try to prevent its progress, and prevent the development of complications [33]. CAM applications in integrative medicine can be defined as diagnosis, treatment, and protection systems that provide a holistic approach to medicine and meet the demands that cannot be met by conventional medicine. In this study, which was performed with the thought that the external application of apple vinegar as an addition to the routine conservative treatment would increase the betterment in symptoms and pain and anxiety levels of patients with varicosity, in compliance with the holistic treatment approach, the expected outcomes were reached (Tables 3 and 4).

Even though some studies examining the effect of vinegar on the cardiovascular system could be found in the literature, no studies examining the effects of the external application of apple vinegar on varicosity could be found [34–38]. Apple cider vinegar contains acetic acid, polyphenols, pectin, and carotenoids with antibacterial and prebiotic properties. Acetic acid is the main ingredient of apple cider vinegar. It is consumable at concentrations of 3–9% [20]. Its antioxidant flavonoid content can reduce the harmful effects of high cholesterol diets [28]. Shishehbor et al. [39] study blood lipid profile of healthy individuals following a high-fat meal that failed to show the positive impacts of apple cider vinegar on serum lipids and lipoproteins. Mansouri et al. [40] found that consuming apple cider vinegar 6% for four weeks improved lipid profiles of healthy and diabetic rats. Setorki et al. [41] detected the benefits of apple cider vinegar consumption on reducing the harmful effects of a high cholesterol diet, including atherosclerotic lesions in the aorta, among rabbits with hypercholesterolemia. Fazelifar and Ghasemi [42] study showed that twelve weeks of aerobic exercise with apple vinegar makes a significant decrease in CRP, cholesterol, and LDL and significantly increased HDL levels. Setorki et al. [43] showed that acute consumption of apple cider vinegar (as an antioxidant) causes significant reduction on some risk factors of atherosclerosis. Fushimi et al. [20] showed that vinegar consumption with diet containing 1% cholesterol for 19 days significantly reduced TG and total cholesterol. Naziroğlu et al. [44] found that consuming apple cider vinegar decreased in lens oxidative injury by modulating GSH-Px in mice fed with high cholesterol. Iizuka et al. [45] found that polyphenols (catechins) present in apple vinegar have the ability to inhibit the LDL oxidation ex vivo in endothelial cells. Budak et al. [27] found a significant reduction in steatosis in the rats treated with apple cider vinegars as oral for 7 weeks when compared to control group. In the literature, studies on CAM applications on varicosity patients regarding hydrotherapy, hirudotherapy, and homeopathy were found and positive effects of CAM methods on symptoms were found in these studies [46–50]. In another study, drinking apple vinegar was stated to decrease obesity [51]. Although not similar to our study, it can be seen in these studies that CAM methods provide positive effects. In our study, the external application of apple vinegar was found to increase the effect of conservative treatment and meaningfully decrease symptoms, pain, and anxiety levels without causing any side effects.

## 5. Conclusion

We determined that the external application of apple vinegar on varicosity patients, which is a very easy application, increased the positive effects of conservative treatment. CAM methods are not definitive treatment methods, but when applied in compliance with scientific medical methods, they can support increasing the quality of life of patients. For this reason, CAM methods widely used within the society should be tested in new scientific studies to prove their effectiveness and evaluate the outcomes of their use alongside medical treatment, which would also provide foresight and contribution to integrative medicine practitioners.

## Figures and Tables

**Table 1:** The distribution of patients according to sociodemographic characteristics (*n* = 80).

Characteristics	Application group		Control group			
	(*n* = 40)		(*n* = 40)		*χ* ^2^	*p*
	*n*	%	*n*	%		
Gender						
Female	32	80.0	30	75.0	2.65	*p* > 0.05
Male	8	20.0	10	25.0		
Education level						
Illiterate	1	2.5	2	5.0	4.11	*p* > 0.05
Literate	5	12.5	7	17.5		
Elementary	22	55.0	24	60.0		
High	9	22.5	6	15.0		
College	3	7.5	1	2.5		
Marital status						
Married	33	82.5	32	80.0	1.56	*p* > 0.05
Single	7	17.5	8	20.0		
Occupation						
Housewife	14	35.0	11	27.5	0.66	*p* > 0.05
Laborer	10	25.0	13	32.5		
Retired	1	2.5	3	7.5		
Officer	8	20.0	9	22.5		
Independent	4	10.0	6	15.0		
Smoking						
Yes	14	35.0	15	37.5	4.55	*p* > 0.05
No	26	65.0	25	62.5		
Varicose veins in the first-degree relative						
Yes	23	57.5	25	62.5	1.66	*p* > 0.05
No	17	42.5	15	37.5		
Alcohol						
Use	5	12.5	7	17.5	3.68	*p* > 0.05
Nonuse	35	87.5	33	82.5		
Age (years)	38.96 ± 11.21		36.96 ± 10.43		*t* = 12.36	*p* > 0.05

Categorical variables were presented as the number (percent); continuous variables were presented as mean ± standard deviation.

**Table 2:** The distribution of patients according to clinic characteristics (*n* = 80).

Characteristics	Application group		Control group			
	(*n* = 40)		(*n* = 40)		*χ* ^2^	*p*
	*n*	%	*n*	%		
Hypertension						
Yes	9	22.5	8	20.0	3.21	*p* > 0.05
No	31	77.5	32	80.0		
CEAP classification						
Class 1	14	35.0	12	30.0	1.98	*p* > 0.05
Class 2	16	40.0	15	37.5		
Class 3	10	25.0	13	32.5		
BMI (kg/m^2^)						
<18.5	4	10.0	2	5.0	1.22	*p* > 0.05
18.5–24.9	9	22.5	11	27.5		
25–29.9	16	40.0	13	32.5		
30–34.9	10	25.0	11	27.5		
35–39.9	1	2.5	2	5.0		
>40	0		1	2.5		
DM						
Yes	6	15.0	4	10.0	2.77	*p* > 0.05
No	34	85.0	36	90.0		

CEAP: C: clinical presentation, E: etiologic factors, A: anatomical distribution, and P: pathophysiological conditions; DM: diabetes mellitus; HT: hypertension; BMI: body mass index.

**Table 3:** Symptoms before and after apple vinegar application (*n* = 80).

	Application group (*n* = 40)		Control group (*n* = 40)	
Symptoms	Before application	After application	Before application	After application
	*n* (%)	*n* (%)	*n* (%)	*n* (%)
Pain				
Slight	8 (%20)	18 (%45)	7 (%17.5)	13 (%32.5)
Disturbing	16 (%40)	13 (%32.5)	19 (%47.5)	17 (%42.5)
Severe	13 (%32.5)	9 (%22.5)	12 (%30)	10 (%25)
Very severe	3 (%7.5)	0 (%0.0)	2 (%5)	0 (%0.0)
	*χ* ^2^ = 18.75	**p** = 0.02	*χ* ^2^ = 8.55	**p** = 0.05
Cramp				
No	12 (%30)	19 (%47.5)	11 (%27.5)	14 (%35)
Sometimes	22 (%55)	19 (%47.5)	24 (%60)	23 (%57.5)
Yes	6 (%15)	2 (%5)	5 (%12.5)	3 (%7.5)
	*χ* ^2^ = 11.87	**p** = 0.04	*χ* ^2^ = 3.98	*p* = 0.08
Edema				
No	11 (%27.5)	21 (%52.5)	12 (%30)	16 (%40)
Slight	25 (%62.5)	19 (%47.5)	25 (%62.5)	24 (%60)
Severe	4 (%10)	0 (%0.0)	3 (%7.5)	0 (%0.0)
	*χ* ^2^ = 22.99	**p** = 0.01	*χ* ^2^ = 4.13	*p* = 0.11
Itching				
No	31 (%77.5)	38 (%95)	32 (%80)	35 (%87.5)
Yes	9 (%22.5)	2 (%5)	8 (%20)	5 (%12.5)
	*χ* ^2^ = 16.78	**p** = 0.02	*χ* ^2^ = 2.44	*p* = 0.09
Pigmentation				
No	28 (%70)	35 (%90)	26 (%65)	24 (%60)
Slight	12 (%30)	4 (%10)	14 (%35)	16 (%40)
Severe	0 (%0)	0 (%0)	0 (%0)	0 (%0)
	*χ* ^2^ = −22.7	**p** = 0.00	*χ* ^2^ = 3.21	*p* = 0.10
Weight feeling				
Slight	9 (%22.5)	15 (%37.5)	9 (%22.5)	12 (%25)
Disturbing	15 (%37.5)	19 (%47.5)	18 (%45)	21 (%52.5)
Severe	12 (%30)	5 (%12.5)	11 (%27.5)	5 (%12.5)
Very severe	4 (%10)	1 (%2.5)	2 (%5)	2 (%5)
	*χ* ^2^ = 10.21	**p** = 0.05	*χ* ^2^ = 2.12	*p* = 0.12
Leg fatigue				
Slight	4 (%10)	18 (%45)	6 (%15)	15 (%37.5)
Disturbing	22 (%55)	17 (%42.5)	21 (%52.5)	22 (%55)
Severe	11 (%27.5)	4 (%10)	12 (%25)	2 (%5)
Very severe	3 (%7.5)	1 (%2.5)	1 (%2.5)	1 (%2.5)
	*χ* ^2^ = 38.23	**p** = 0.00	*χ* ^2^ = 12.51	**p** = 0.04

**Table 4:** The pain perception and social appearance anxiety score averages of the patients (*n* = 80).

	Application group (*n* = 40)		Control group (*n* = 40)	
	Before application	After application	Before application	After application
	Ort. ± SS	Ort. ± SS	Ort. ± SS	Ort. ± SS
SAAS	41.90 ± 10.96	35.67 ± 9.88	40.69 ± 11.41	36.56 ± 10.75
	*t* = 3.12	**p** = 0.00	*t* = 2.45	**p** = 0.05

VAS	5.21 ± 2.56	4.15 ± 2.93	5.02 ± 2.31	4.75 ± 2.11
	*t* = 3.02	**p** = 0.00	*t* = 1.45	**p** = 0.05

SAAS: Social Appearance Anxiety Scale, VAS: Visual Analog Scale.
